# A Cell Motility Screen Reveals Role for MARCKS-Related Protein in Adherens Junction Formation and Tumorigenesis

**DOI:** 10.1371/journal.pone.0007833

**Published:** 2009-11-18

**Authors:** Alexander E. Finlayson, Kevin W. Freeman

**Affiliations:** 1 Department of Genetics, Harvard Medical School, Boston, Massachusetts, United States of America; 2 Vascular Biology Program, Department of Surgery, Children's Hospital, Boston, Massachusetts, United States of America; University of Helsinki, Finland

## Abstract

Invasion through the extracellular matrix (ECM) is important for wound healing, immunological responses and metastasis. We established an invasion-based cell motility screen using Boyden chambers overlaid with Matrigel to select for pro-invasive genes. By this method we identified antisense to MARCKS related protein (MRP), whose family member MARCKS is a target of miR-21, a microRNA involved in tumor growth, invasion and metastasis in multiple human cancers. We confirmed that targeted knockdown of MRP, in both EpRas mammary epithelial cells and PC3 prostate cancer cells, promoted *in vitro* cell migration that was blocked by trifluoperazine. Additionally, we observed increased immunofluoresence of E-cadherin, β-catenin and APC at sites of cell-cell contact in EpRas cells with MRP knockdown suggesting formation of adherens junctions. By wound healing assay we observed that reduced MRP supported collective cell migration, a type of cell movement where adherens junctions are maintained. However, destabilized adherens junctions, like those seen in EpRas cells, are frequently important for oncogenic signaling. Consequently, knockdown of MRP in EpRas caused loss of tumorigenesis *in vivo*, and reduced Wnt3a induced TCF reporter signaling *in vitro*. Together our data suggest that reducing MRP expression promotes formation of adherens junctions in EpRas cells, allowing collective cell migration, but interferes with oncogenic β-catenin signaling and tumorigenesis.

## Introduction

Invasion of cells through the extracellular matrix (ECM) is a complicated process involving migration by two alternating modes of action with either cellular deformation allowing movement through the ECM or by degradation of the ECM using factors such as MMPs [1], [2]. An established approach for assaying cellular movement through the ECM is using Boyden chambers overlaid with Matrigel, a basement membrane extract composed of extracellular matrix. Though Matrigel lacks many of the important attributes of endogenous basement membrane, such as less physical cross-linking [3], it is frequently used to identify chemical factors that either promote or inhibit invasion of a particular cell type. Since movement of cells through the extracellular matrix (ECM) is necessary for normal physiological processes, such as wound healing and movement of immune cells, in addition to pathological processes, such as metastasis [4], we decided to establish an *in vitro* invasion-based cell motility genetic screen.

Using the Boyden chamber assay with Matrigel we selected for and identified antisense to MARCKS-related protein (MRP), a member of the MARCKS family of proteins. MARCKS family members have been implicated in actin cytoskeletal regulation, the protein kinase C (PKC) signaling pathway and the calmodulin (CaM) signaling pathway [5]. MARCKS family members have strong affinity for calcium-calmodulin (Ca2+−CaM), but do not bind to CaM [6], [7]. Additionally they are abundantly expressed making them a possible reservoir of Ca2+−CaM signaling [7], [8]. Important to their regulation MRP and MARCKS are capable of reversibly binding to the plasma membrane through the combination of their N-terminal myristoylation group and their effector domain (ED). At the plasma membrane MARCKS/MRP are thought to bind and sequester phosphatidylinositol bisphosphate (PIP2) through the strongly basic ED. Phosphorylation of the ED by protein kinase C (PKC) prevents binding of MARCKS/MRP to the plasma membrane and to Ca2+−CaM. Binding of Ca2+−CaM to the ED also prevents binding of MARCKS/MRP to the plasma membrane and blocks phosphorylation by PKC. This mutual exclusive relationship suggests that MARCKS family members integrate PKC and CaM signaling [9], [10]. Furthermore the sequestration of PIP2 by MARCKS regulates accessibility to this important signaling substrate [11]. Based on these interactions this places MARCKS family members at the nexus of a number of critical signaling pathways in cancer. Recently MARCKS was shown to be a target of miR-21 a micro-RNA that promotes invasion and metastasis in a number of human cancers implicating it as an important tumor suppressor [12]. In this report through an invasion-based motility screen we identified MRP as affecting adherens junction formation and tumorigenesis of EpRas cells.

## Materials and Methods

### Tumor Cell Lines and Constructs

EpH4 and EpRas cells were maintained in complete media (DMEM with 10%FBS). PC3, HeLa, HCT-116 and MCF-7 cells were originally obtained from ATCC and maintained in complete media. EpRas cells were obtained from Martin Oft (Schering-Plough Biopharma, Palo Alto, California). Pooled stable cell lines were selected in 3 µg/mL puromycin for two weeks after EpH4 cells were transfected with either p-Babe-puro or p-Babe-puro-asMRP. EpRas clonal lines were selected in 3 µg/mL puromycin for two weeks after transfection with control shRNA or shRNAs to human and mouse MRP (Open Biosystems), colonies were picked and maintained in media with puromycin. To make the p-Babe-puro-asMRP construct an *EcoRI* containing asMRP fragment of pEyk3.1-asMRP isolated from the screen was subcloned into the *EcoRI* site of p-Babe-puro.

### Real Time Quantitative PCR and Northern

MRP mRNA expression as assessed by Northern analysis using P^32^ 5′-labeled DNA probes to MRP and GAPDH by methods previously described [13], quantitated with STORM phosphorimager and normalized to GAPDH. MRP mRNA levels were analyzed by qt-PCR using SYBR green and normalized to actin. qt-PCR was performed using primer pairs (TTCTTTTCCAAGTAGGTTTTGTTTACC and CACTCAAGGTTTGGGAGTATAAGCA) for MRP and primer pairs (GCCAACCGC-GAGAAGATGA and CATCACGATGCCAGTGGTA) for actin.

### Retroviral Generation and Infections

Procedures were followed as described in greater detail in Koh et. al. [14]. Briefly 293T cells were transfected at a 1∶1 ratio of the retroviral construct to the packaging construct PCL-eco using FuGENE6 (Roche). The medium was changed 16 hours after transfection. At 30 hours after transfection, supernatants were isolated and cells were spin infected with 8 µg/ml of polybrene (Sigma).

### Cell Migration and Invasion-Based Motility Assay

Invasion-based motility assays and screen were performed with BD BioCoat Matrigel invasion chambers (BD Biosciences). Cell migration assay was performed using 8.0-µm pore size 96-well MIC Transwell plates (Millipore). For both procedures cells were serum-starved overnight, harvested with TrypLE Express (Invitrogen), and washed twice with serum-free DMEM. Cells were then re-suspended in DMEM and were added to the upper chamber while the lower chamber was filled with complete media as a chemoattractant. After 16 hours at 37°C, the cells on the upper surface of the membrane were removed by cotton tips. For the screen, cells were harvested using TrypLE Express and cell scraping then allowed to recover in complete media for 48 hours prior to additional rounds of selection. For migration and invasion-based motility assay, cells attached to the lower surface were fixed in ice-cold methanol for 10 minutes, and stained for 10 minutes with a solution containing 0.5% crystal violet and 2% ethanol in 100 mM borate buffer (pH 9.0). The number of migrated cells on the lower surface of the membrane was counted under a microscope in five fields (100×). For wound healing assay cells were plated onto 35 mm gridded dishes (Ibidi). Confluent cells where wounded with 100 uL tip and monitored for migration into the wound.

### Luciferase Reporter Assay

Indicated cells were plated onto 12-well plates and transfected with 400 ng TOPFLASH (Millipore) β-catenin reporter construct with firefly luciferase and as a transfection control, 100 ng pRL-CMV (Promega) construct with renilla luciferase. Cells were treated with Wnt3a (R&D systems) for 24 hours starting 24 hours after transfection. Luciferase was assayed using dual-luciferase reporter assay system (Promega) on a TD-20/20 luminometer (Turner Biosystems) using manufacturer instructions.

### Tumor and Tail Vein Metastasis

EpRas cells were injected intravenously with 5×10^5^ cells in 0.1 ml PBS via tail veins or subcutaneous. All tissue was fixed in buffered formalin and than store in 70% ethanol. After washing with fresh PBS, fixed tissues were dehydrated, cleared, and embedded in paraffin. Sections (5 µm) were collected on microscope slides, deparaffinized, and stained with H & E as routine procedures.

### Western Blots and Immunofluoresence

For immunofluoresence, cells were plated on glass cover slips, serum starved overnight, formaldehyde fixed and permeabilized with methanol for immunofluorescence and imaged at 100X oil-immersion. Cells were incubated with primary antibodies to E-cadherin (Becton Dickinson), APC (Calbiochem) or β-catenin (Becton Dickinson) and secondary antibodies Alexa fluor 488 anti-rabbit or anti-mouse (Invitrogen). For western blots, cells were lysed was with MPER (Pierce) and run on SDS-Page immunoblotted with antibodies to Ras (Cell Signaling), E-cadherin (Becton Dickinson), β-catenin (Becton Dickinson), or γ-tubulin (Sigma), followed by secondary mouse or rabbit HRP (Amersham Biosciences).

### Statistical Analysis

Data were expressed as mean±SD. Student's *t*-test was used to evaluate the difference between two groups. P<0.05 was considered to be significant.

## Results

### An Invasion-Based Motility Screen Identifies Antisense to MRP

**Figure 1:**
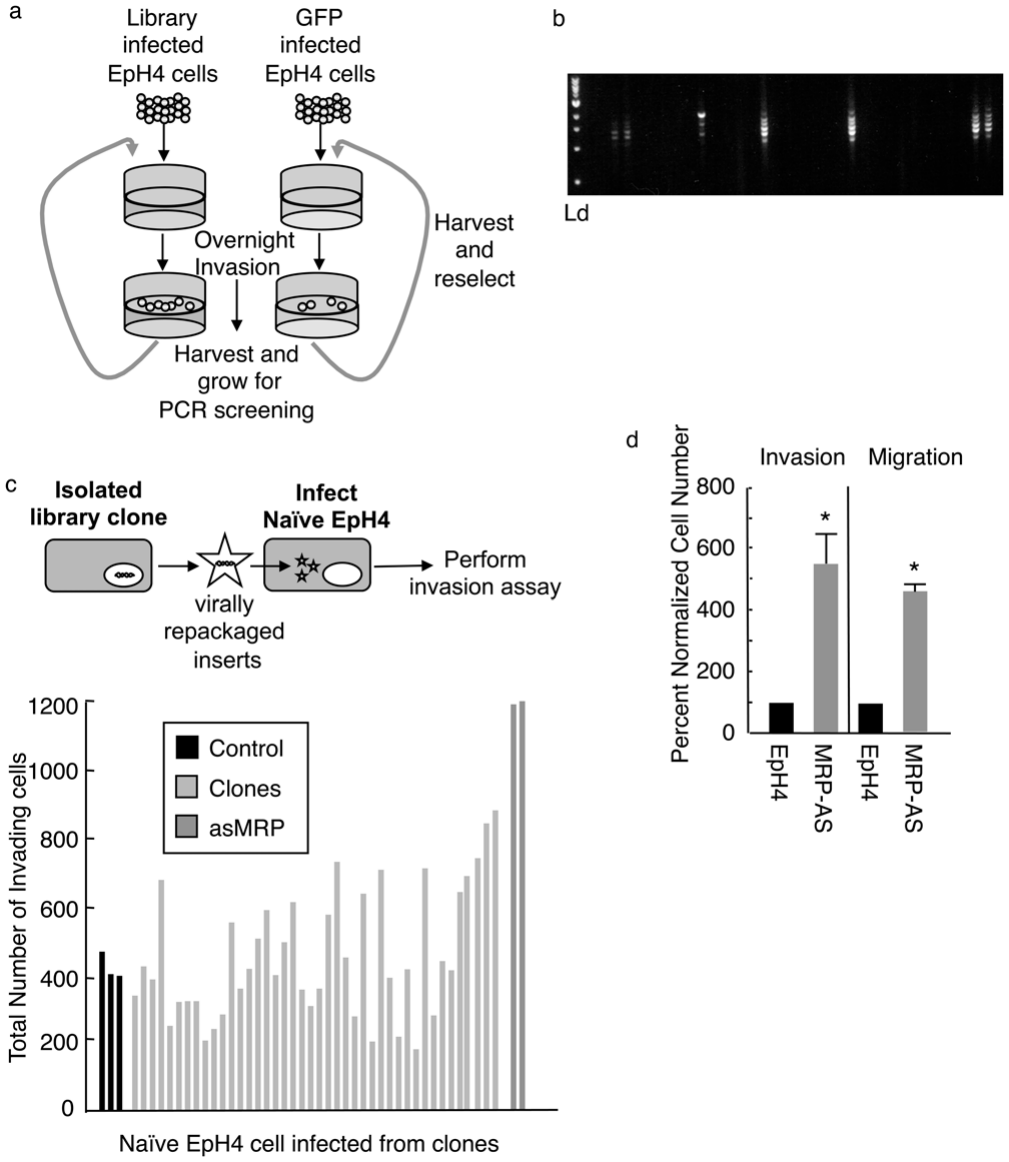
Isolation of antisense to MRP in Invasion-based motility screen. a) Schematic of ECM screen. b) Representative genomic PCR of isolated clones for inserts in pEyk3.1, Ld =  1 kb ladder (NEB). c) To segregate multiple retroviral integrants, retrovirus was repackaged from either invading clones or the control EGFP clones using pCL-Eco helper plasmid (top schematic). Naive EpH4 cells were infected and individual clonal lines were assayed for invasion. The two most invasive clones were determined to have both been infected with antisense to MRP (dark grey). d) Mouse mammary cell line EpH4 was stably transfected with expression pβ plasmid containing antisense MRP identified in the screen or control plasmid and subjected to invasion or migration assays. Data is mean of three independent experiments ± SD. * is p<0.01

We established a novel strategy to select for genes that promote *in vitro* motility through Matrigel, a basement membrane extract composed of extracellular matrix. For our screen the immortalized mouse mammary cell line EpH4 was chosen to better reflect normal mammary epithelial cells. We transiently transfected 293 cells with a pEyk3.1 retroviral cDNA library made from mRNA isolated from mouse embryos or a control pEyk3.1 retrovirus containing EGFP [14]. We harvested viral supernatant from the 293 cell cultures and infected 6×10^6^ EpH4 cells with either the library or EGFP retroviral particles. Both library and EGFP infected EpH4 cells were screened in parallel. The selection protocol involved plating cells starved for 18 hours onto Boyden chambers overlaid with Matrigel. We then allowed cells to invade overnight using complete media as a chemoattractant. Invasive cells were harvested, plated in complete media and recovered for 48 hours before another round of selection (Fig. 1a). After three rounds of selection we observed an increase in library infected cells over control, therefore cells were harvested, clonally grown and analyzed by genomic PCR for retroviral integrants (Fig. 1b). This analysis revealed 49 out of 500 positive clones.

To confirm promotion of migration each of the 49 clones were individually transfected with a pCL-Eco helper plasmid for repackaging retroviral integrants and subsequent infection of naïve EpH4 cells (Fig. 1c). Newly infected EpH4 cells were then submitted to the same Boyden chamber assay used in the screen. We observed two wells showing an increase of approximately 3 fold above mock-infected cells (Fig. 1c) suggesting at least a subset of genes was conferring increased movement through Matrigel. The genes identified in the two positive clones were antisense to Marcks-related protein (MRP), C-terminally truncated potassium channel KCC4 (ctKCC4) and antisense to ODC-AZ (asODC-AZ).

Since we observed multiple retroviruses transferring into the naïve EpH4 cells, we tested each individually to determine which were responsible for increased migration through Matrigel. To determine this asMRP, ctKCC4 or asODC-AZ were individually cloned downstream of the chicken beta-actin promoter into the puromycin resistance carrying pBabe-puro vector. After selection under puromycin we tested these pooled stable cell lines for increased movement through Matrigel, identifying antisense to MARCKS related protein (asMRP) as conferring approximately a five-fold increase in migration and invasion in comparison to pBabe control pooled stables (Fig. 1d).

### MRP-shRNA Induces Migration in EpRas Cells

**Figure 2:**
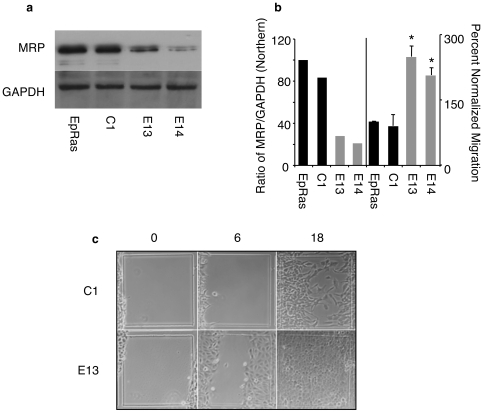
Targeted knockdown of MRP in EpRas promotes migration. a) Parental EpRas cells, clonal cell lines stably transfected with control shRNA (C1) or with shRNA (Open Biosystems) directed to mouse MRP (E13, E14). a) and left panel of b) Quantified MRP mRNA expression as assessed by Northern blot analysis and normalized to GAPDH (Northern shown). Right panel of b) The cell lines were assayed for transwell migration and normalized to EpRas, * is p<0.01 c) Confluent cells of C1 and E13 were assayed for migration into wound at 0, 6, and 18 hours after wounding. Note migration from left grid line as reference. Representative of three experiments.

Since EpH4 cells are a non-tumorigenic cell line we were interested in using the tumorigenic v-HA-Ras transformed EpH4 cells (EpRas) for *in vivo* studies and we chose to knockdown gene expression with MRP shRNA. Parental EpRas cells, control EpRas, which had undergone clonal selection with puromycin after transfection with shRNA to GFP (C1), or shRNA targeted knockdown of MRP and clonal selection by puromycin (E13, E14) were tested for loss of MRP expression by Northern blot analysis. MRP mRNA expression of control (C1) cells was 80%, EpRas13 (E13) clone was 33% and EpRas14 (E14) clone was 20% of parental EpRas cells (Fig. 2a and b). When normalized to migration of EpRas both E13 and E14 showed 2-fold or greater increase in migration (Fig. 2b). We also used a wound-healing assay to test the effects of targeted knockdown on migration. Both E13 and E14 had faster wound healing with cells showing collective migration into the wound, while EpRas and C1 had delayed migration with cells loosely associating (Fig. 2c and Fig. S1). Single cell migration is a well-studied mode by which cells invade their surroundings and frequently cancer cells acquire this ability through mechanisms such as epithelial-mesenchymal transition (EMT). However less understood is collective cell migration where cells move without loss of cell-cell attachments. Collective cell migration is frequently observed in many invasive tumors and is developmentally important for organogenesis [15]–[17]. By both migration assays, reduced MRP increased migration of EpRas cells. The wound-healing assay also implicates a role in collective migration, since cell-cell associations did not impede migration.

### MRP-shRNA Increased *In Vitro* Migration That Is Blocked by TFP

**Figure 3:**
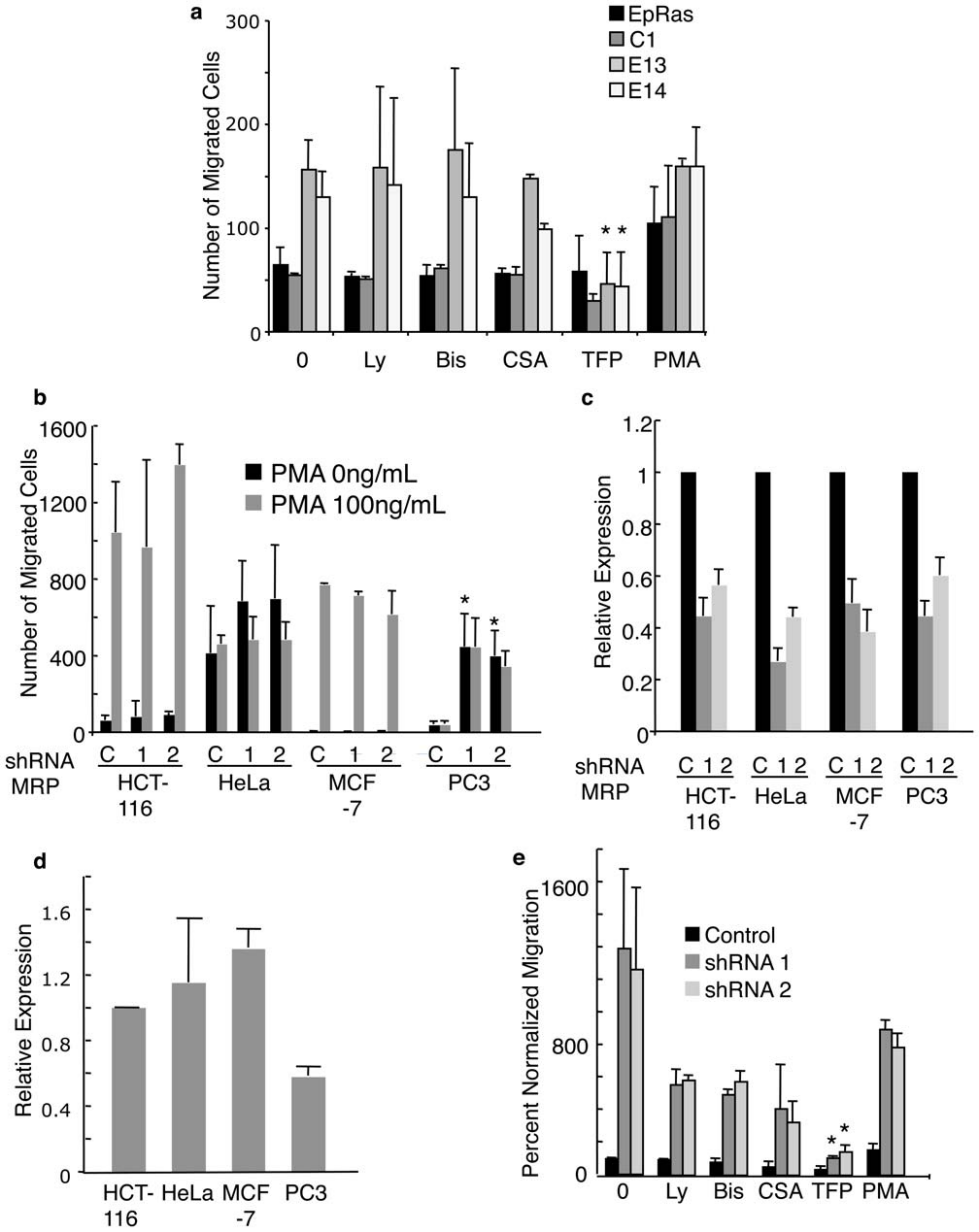
Effects of MRP targeted knockdown on migration is blocked by trifluoperazine. a) Parental EpRas cells, C1, E13 and E14 were assayed for transwell migration with or without chemical inhibitors (Ly294002 50 µM, Bis 1 µM, CSA 10 ug/mL, TFP 50 µM, PMA 100 ng/mL). The number of cells that migrated to the bottom of the transwell after 16 hours were counted and normalized to control, * is p<0.05 b) Indicated human cell lines were tranfected with shRNA to GFP (C) or two different shRNAs (1,2) to human MRP (Open Biosystems) and analyzed by transwell assay, * is p<0.01. c,d) MRP mRNA levels were analyzed by qt-PCR and normalized to actin after transfection with control shRNA or shRNAs to human MRP (1,2) (c) or basal MRP mRNA levels (d). e) PC3 cells were transfected with shRNA1 or 2 and analyzed as in (a), * is p<0.05. All data is mean of three independent experiments ± SD.

Three possible methods of regulating migration by MRP are the direct regulation of the cytoskeleton through actin, sequestration of PIP2 and thereby regulating PKC mediated cell migration and lastly, functioning as a reserve for Ca2+/CaM and therefore regulating its control of migration [7], [8]. To determine which of these pathways is promoting migration in EpRas cells we used inhibitors to PI-3K (Ly294002), PKC (bisindolylmaleimide), calcineurin (Cyclosporin A), and calmodulin (trifluoperazine) as well as an activator of PKC (Phorbol 12-myristate 13-acetate). Only the CaM inhibitor trifluoperazine (TFP) reduced migration of E13 and E14 to EpRas migration levels while not appreciably affecting the migration of EpRas or C1 (Fig. 3a). This suggests that the calmodulin (CaM) pathway may be important for MRP knockdown induced migration in EpRas cells. One cautionary note is the effect of calmodulin inhibitors, such as TFP, on electrostatic surface potential, which could interfere with multiple pathways in addition to calmodulin [18].

To determine if MRP knockdown would also promote migration in other cell lines we transiently transfected a panel of human cancer cell lines with two different human shRNAs to MRP or control shRNA to GFP. We tested the colon cancer line HCT-116, the cervical carcinoma line HeLa, the mammary adenocarcinoma MCF-7, and the prostate cancer line PC3. In our Boyden migration assay serum containing media was used as a chemoattractant and PMA was added as a positive control to demonstrate poorly migrating cells were competent for migration. PC3 cells showed an approximate 12-fold increase in migration when transiently transfected with shRNAs to MRP, which was not improved by addition of PMA (Fig. 3b). By quantitative PCR we showed an approximate 50% reduction of MRP mRNA levels in all four cell lines tested 48 hours after transient transfection with MRP shRNA 1 or 2 (Fig. 3c and d). We also determined differences in MRP expression between the cell lines and noted that PC3 has the lowest starting level of MRP, being 60% of HCT-116 levels and 50% or less of HeLa and MCF-7 (Fig. 3d). We used the same chemical inhibitor approach taken with EpRas to determine what pathway was mediating the migration of PC3 cells. Unlike EpRas cells other inhibitors showed modest but not statistically significant reduction in PC3 migration induced by shRNA to MRP. However similar to EpRas cell, PC3 cells transfected with shRNA to MRP were most strongly inhibited by TFP (Fig. 3e).

### Loss of MRP Causes Establishment of Adherens Junctions and Reduced β-Catenin Signaling

**Figure 4:**
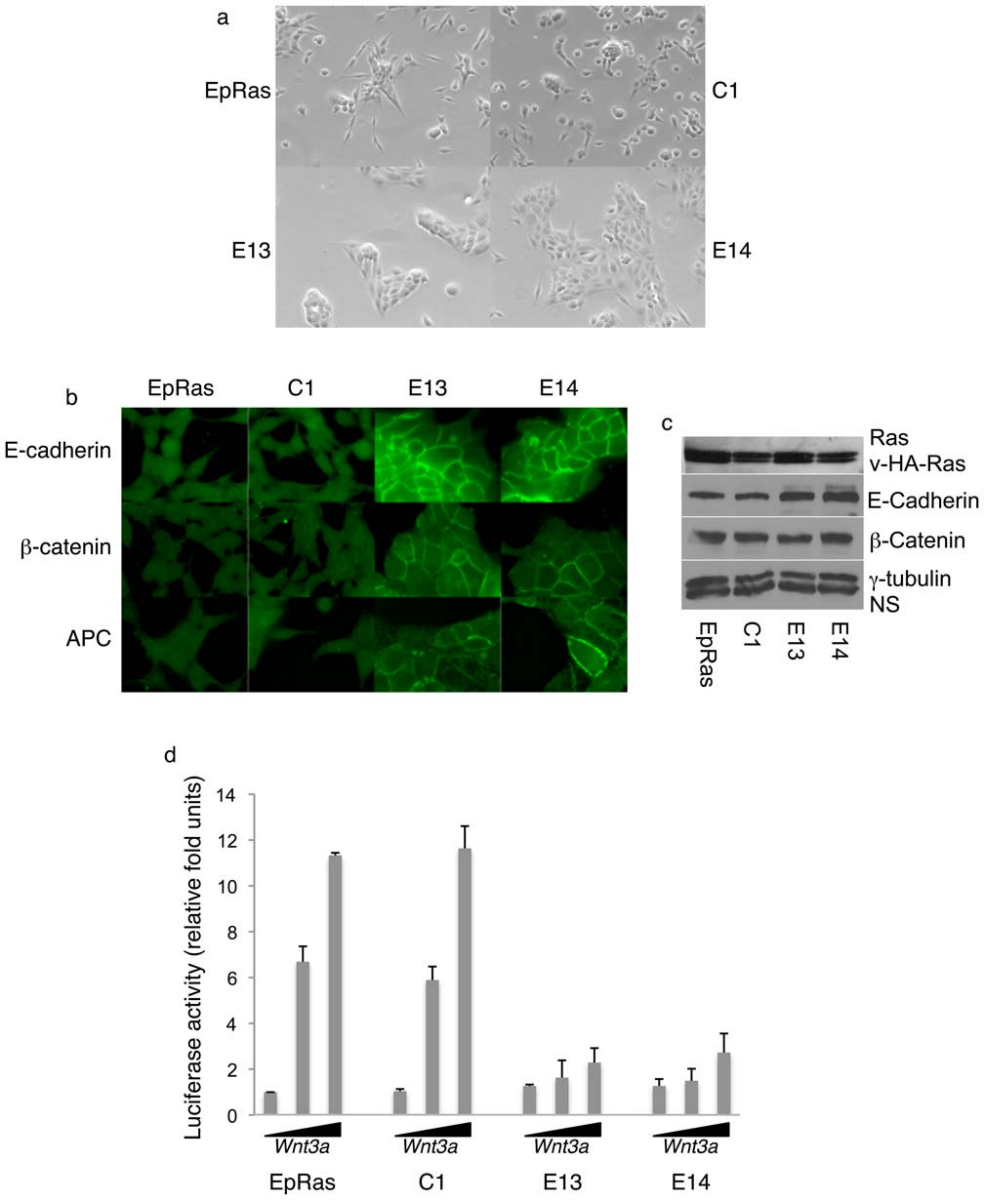
Targeted knockdown of MRP leads to establishment of adherens junctions. a) Bright field imaging of EpRas, C1, E13, and E14 at 10X. b) EpRas, C1, E13 or E14 cells were stained with primary antibodies specific to either E-cadherin (Becton Dickinson), APC (Calbiochem) or β-catenin (Becton Dickinson) and secondary antibodies Alexa fluor 488 anti-rabbit or anti-mouse (Invitrogen). Images at 100X c) Western blot analysis of EpRas, C1, E13, E14 whole cell lysates and immunoblotted with antibodies to Ras (Cell Signaling), E-cadherin and β-catenin (Becton Dickinson), and γ-tubulin (Sigma). NS =  non-specific. d) Indicated cells transfected with the TCF luciferase reporter construct TOPFLASH and transfection control pRL-CMV were treated with 0, 50 ng/mL or 100 ng/mL of recombinant WNT3a for 18 hours. All data is mean of three independent experiments ± SD.

EpRas cells with MRP knockdown (E13, E14) showed increased cell clustering suggesting establishment of adherens junctions (Fig. 4a). When directly comparing cells in clusters, we observed an increased immunofluoresence for the adherens junction markers E-cadherin, APC and β-catenin in MRP-knockdown cells at sites of cell-cell contact (Fig. 4b). We wanted to determine if E-cadherin levels were affected by changes in adherens junctions so we analyzed by western blot the expression levels of both E-cadherin and β-catenin, observing increased E-cadherin expression but no changes in β-catenin in MRP-knockdown cells (Fig. 4c). We next wanted to determine if formation of adherens junctions would affect β-catenin signaling since catenins are known to interact with the cytoplasmic domain of E-cadherins at sites of adherens junction. With loss of adherens junctions and in response to Wnt signaling, β-catenin binds to TCF-LEF-1 family of transcription factors to activate gene expression [19]. We used the TCF-luciferase reporter TOPFLASH to assess the role of MRP in β-catenin signaling. While E13 and E14 show a two-fold induction of reporter activity, an approximate 12-fold induction was seen for both EpRas and C1 in response to Wnt3a (Fig. 4d).

### Loss of MRP Causes Loss of Tumorigenecity

**Figure 5:**
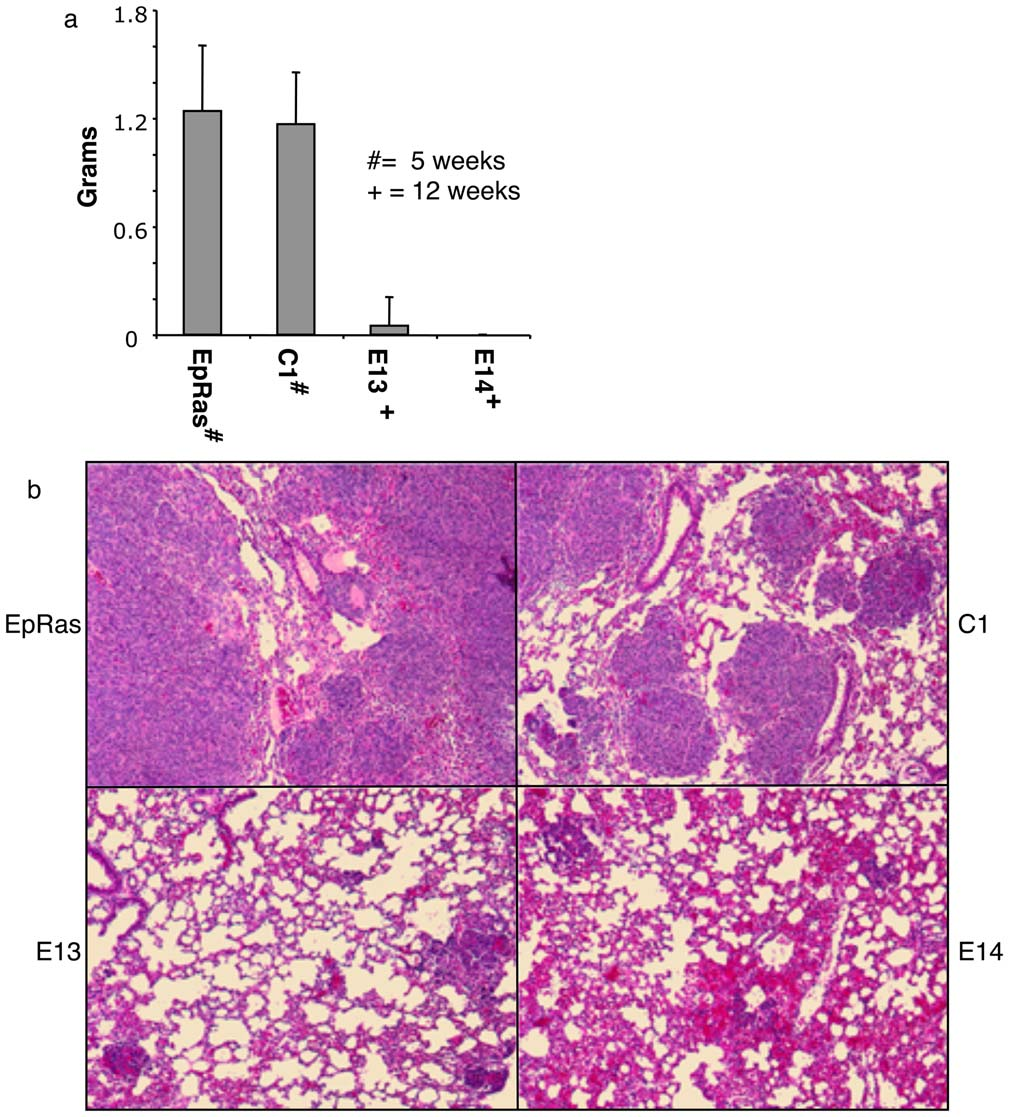
Loss of tumorigenesis with targeted knockdown of MRP. 5×10^5^ cells EpRas cells, clonal cell lines stably transfected with control shRNA (C1) or with shRNA directed to MRP (E13, 14) were injected subcutaneously into five congenic Balb/c mice per cohort (a) or into 10 mice per cohort by tail vein injection (b). Tumor weight was assessed at the indicated times. Representative H&E staining of lung sections from each group at 4× magnification (b).

An increase in adherens junctions and reduced β-catenin signaling could interfere with tumor progression, however the effects of reduced MRP on cellular invasion may increase local invasion and metastasis. We carried out experiments to determine the effects of reduced MRP on both tumorigenesis and metastasis since no previous *in vivo* cancer studies had been performed with MARCKS family members. For both subcutaneous and tail vein injections we injected 5×10^5^ of EpRas, C1, 13 or 14. Cumulative of two independent experiments both EpRas and C1 showed rapid subcutaneous tumor development with ten out of ten mice for EpRas having average tumor weights of 1.4 grams and six out six mice for C1 having an average of 1.25 grams for C1 by 4 weeks, while no tumors out of ten mice grew out for E14 and only one out of ten mice developed a tumor in the E13 (Fig. 5a). The single E13 tumor required 12 weeks to achieve 0.5 gram and the mouse was sacrificed at this smaller tumor size due to ulceration at the tumor site. Histological analysis of the lungs and livers of all the animals showed no evidence of any metastasis. For intravenous tail vein injections, again as cumulation of two independent experiments, ten mice were injected per cohort with EpRas and C1 showing aggressive tumor numbers and size in the lungs while E13 and E14 had far fewer tumors. The mets that did develop for E13 and E14 were much smaller in size when mice were sacrificed 4 weeks after tail vein injections as represented in Figure 5b. We were concerned that the observed loss of tumorigenecity could be due to loss of the transforming v-HA-Ras during clonal selection. Western blot analysis revealed no obvious difference in either endogenous or v-HA-Ras expression between the parental EpRas, C1, E13 or E14 (Fig. 4c). Based on the failure of subcutaneous tumors to grow, the greatly reduced number of metastasis observed in the MRP knockdown clones is likely a consequence of lost tumorigenecity.

## Discussion

Our data suggest that knockdown of MRP promotes adherens junction formation. A possible route that MRP could regulate adherens junction formation is through its regulation of calmodulin (CaM) via the scaffolding protein IQGAP. MRP is known to bind to Ca2+/CaM with nanomolar affinity, but does not bind to CaM [6], [7]. IQGAP, which is regulated by Ca2+/CaM is important for controlling cell migration and adherens junction formation [20], [21], making it a possible downstream effector of MRP. Alternatively, in *Drosophila* a novel pathway to adherens junction formation was identified. It involved localization of a synaptotagmin-like protein Btsz to PIP2. Btsz binds PIP2 and increases adherens junction formation through stabilization of E-cadherin. In this system decreasing PIP2 levels blocked adherens junction formation [22]. Interestingly, MARCKS family members regulate accessibility to PIP2 and any reduction in their expression could promote adherens junction formation through this pathway. Whatever may be the case identifying how MRP regulates adherens junction formation is an important next step.

Regulation of adherens junction is also important for controlling tumorigenesis. It has previously been shown that the introduction of wild type APC into the colorectal cancer cell line SW480, which has mutant APC, caused relocalization of the adherens junction proteins β-catenin and E-cadherin to the plasma membrane and inhibited SW480 tumorigenesis [23] similar to what we observed with reduced MRP. Other groups have shown the converse with disruption of adherens junction promoting tumorigenesis and metastasis [24]. These studies all implicate β-catenin signaling as important in tumorigenesis and our data suggest reduced MRP can lead to reduced β-catenin signaling. In cancer, frequently mutations arise that lead to disruption of adherens junctions, such as loss of APC, loss of E-cadherin or mutationally activated β-catenin. It is possible that reduced MRP would promote metastasis without interfering with tumorigenesis in such instances. Using cell lines that are not dependent on the β-catenin pathway for transformation may allow modeling the role of MRP knockdown in cancer invasion and metastasis, *in vivo*.

Our results are somewhat counterintuitive having proteins that promote migration while also promoting adherens junction formation. However this is exactly what occurs with collective migration. For invasion and metastasis both individual and collective migration of cancer cells is observed, with epithelial-mesenchymal transition (EMT) being a common mechanism for individual cell migration, while the mechanisms for collective migration are poorly understood [1], [25]. Prodoplanin, a mucin-like protein found at the leading edge of multiple tumor types, induces tumor cell invasion in the absence of epithelial-mesenchymal transition (EMT) by increasing collective cell migration [26]. Additionally, in the squamous cell carcinoma cell line A431, siRNA to p120-catenin caused loss of adherens junctions but also a 75% reduction in cell invasion. It was established that the cells invaded collectively, which was only possible with p120 maintenance of adherens junctions. Interestingly, squamous cell carcinoma of the head and neck also appears to invade collectively in clinical samples [27]. The importance of collective cell migration in metastasis is becoming of increasing interest and exploration of MARCKS family members in collective cell invasion should be further explored. Additionally MARCKS family members may provide insight into how initiation of cancer and its maintenance differs between metastatic cancers that display collective cell migration behavior and those that undergo EMT. Overall, in this study we established a novel invasion-based cell motility screen identifying MRP and showing its importance in migration, transformation and adherens junction formation.

## Supporting Information

Figure S1 Confluent cells of EpRas,C1, E13, and E14 were assayed for migration into wound at 0, 6, and 18 hours after wounding.
(8.79 MB TIF)
