# Supplementary figures and images for: A Cell Motility Screen Reveals Role for MARCKS-Related Protein in Adherens Junction Formation and Tumorigenesis
Source: PLoS One. 2009 Nov 18;4(11):e7833. doi: 10.1371/journal.pone.0007833 (PMC2774968; doi:10.1371/journal.pone.0007833)

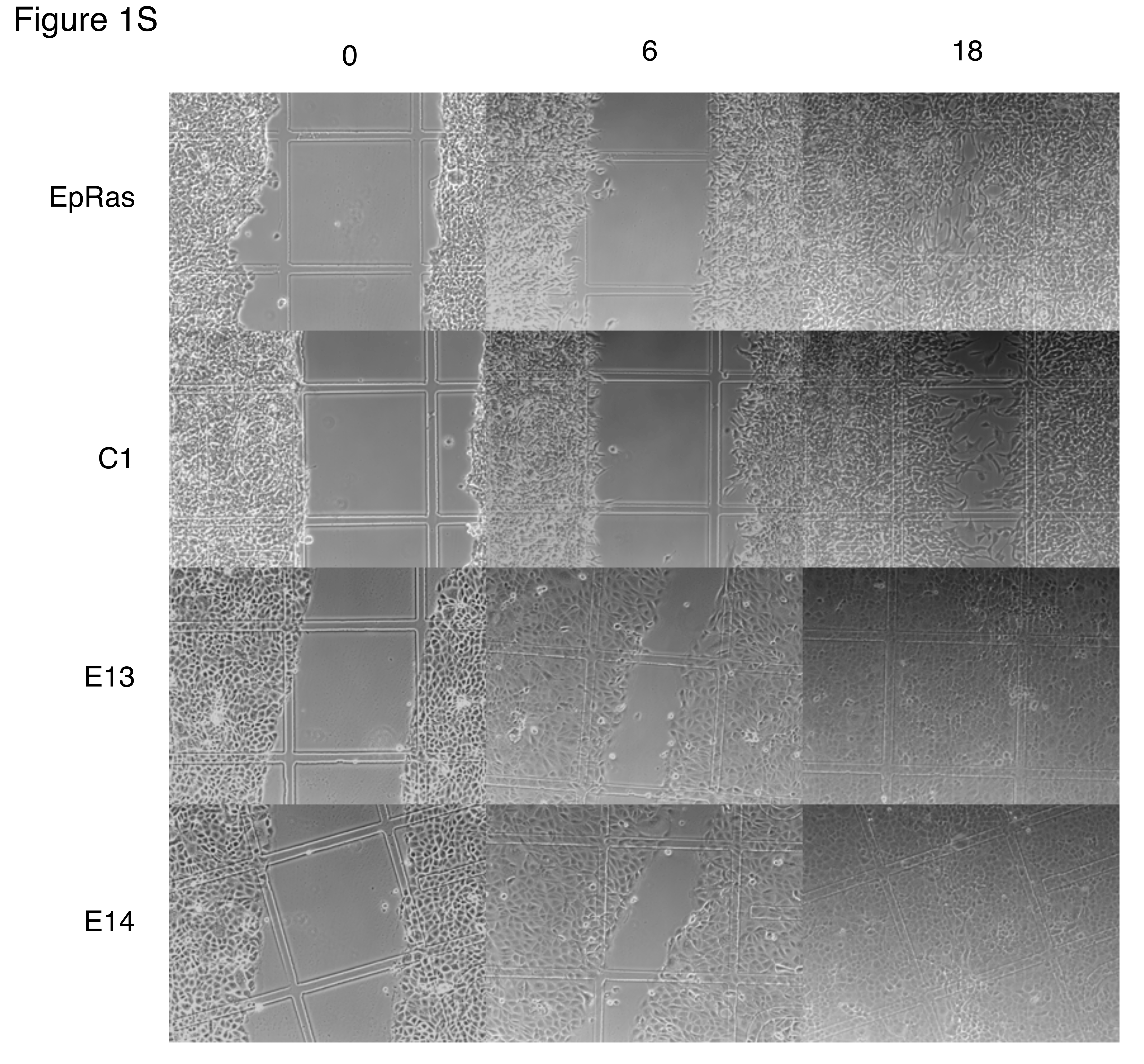

Supplement: Figure S1 — Confluent cells of EpRas,C1, E13, and E14 were assayed for migration into wound at 0, 6, and 18 hours after wounding. (8.79 MB TIF) [file pone.0007833.s001.tif]
